# Diastolic function from tagged MRI and myocardial fibrosis: the Multi-Ethnic study of Atherosclerosis (MESA)

**DOI:** 10.1186/1532-429X-15-S1-O82

**Published:** 2013-01-30

**Authors:** Bharath Ambale Venkatesh, Anderson C Armstrong, Sirisha Donekal, Yuan Chang Liu, Chia-Ying Liu, Kihei Yoneyama, Colin O Wu, Marcelo Nacif, Antoinette S Gomes, W Gregory Hundley, David A Bluemke, Joao A Lima

**Affiliations:** 1 Radiology, Johns Hopkins Hospital, Baltimore, MD, USA; 2 Radiology and Imaging Sciences, National Institutes of Health, Bethesda, MD, USA; 3 Department of Cardiology, Wake Forest University Health Sciences, Winston-Salem, NC, USA; 4 Department of Radiology, UCLA School of Medicine, Los Angeles, CA, USA; 5 National Institutes of Health, Bethesda, MD, USA; 6 Cardiology, Johns Hopkins Hospital, Baltimore, MD, USA

## Background

MRI-derived T1 times are associated with diffuse interstitial myocardial fibrosis. LV diastolic dysfunction and fibrosis are important components of cardiac disease, but their relationship has not been firmly established. Early diastolic strain rate (Epeak) has been validated as an indicator of diastolic function by tagged MRI. We derive a novel strain relaxation index (SRI) based on circumferential strain from tagged MRI to indicate early phases of diastolic dysfunction. SRI is defined as the ratio of very early myocardial relaxation time and tissue compliance. We will assess the relationship between diastolic dysfunction assessed by both SRI and Epeak with myocardial fibrosis.

## Methods

**Figure 1:**
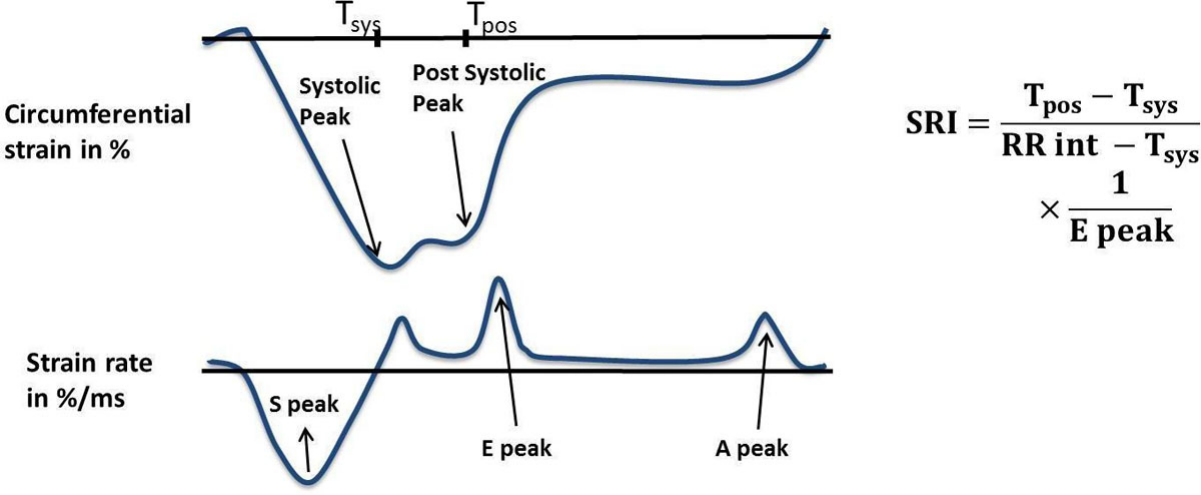
Illustration of the calculation of the proposed SRI from the circumferential strain (top) and strain rate curves (bottom). More negative strain values indicate greater circumferential shortening.

We included MESA participants who underwent MRI examination (delayed gadolinium enhancement, T1 mapping, and cardiac tagging) without visual myocardial replacement scar between 2010 -2012. Harmonic phase analysis was used to compute mid-ventricular circumferential strains and strain rates (Figure 1). SRI was calculated as the difference between post-systolic and systolic times of the strain peaks (indicator of very early myocardial relaxation), divided by the Epeak (a measure of tissue compliance); normalized by the total relaxation time (difference between the RR interval and the systolic interval). Greater SRI values indicated greater diastolic dysfunction. To assess myocardial fibrosis, mid ventricular short-axis T1 maps at pre- and 12' post-contrast injection were acquired using the MOLLI sequence. Linear regression evaluated the cross-sectional relationship between post-contrast T1 times and, SRI and Epeak (both in logarithmic scale). The models included adjustments for covariates that affect T1 times (model 1 = glomerular filtration rate, pre-contrast T1 times adjusted to the heart rate, time difference between gadolinium injection and imaging, and gadolinium dosage in mmol/kg) and demographic parameters (model 2 = model 1 + age, gender, ethnicity).

## Results

1080 participants (52.7% women, mean age 67±9 years, 51% Caucasian, 12% Chinese-American, 22% African-American, 15% Hispanic) were considered and 97 excluded due to the presence of myocardial scar. Mean values for logSRI, logEpeak and T1 (12min) were 0.95±0.58 s, 0.11±0.04 /s and 455±40 ms respectively. SRI and post-contrast T1 times were inversely related (Table 1). This relation remained significant after adjustment for covariates in models 1 and 2. Epeak was not related to post-contrast T1 times in any of the models.

**Table 1:** Coefficients (p-values) for linear regression between of SRI and Epeak (in logarithmic scale), and post-contrast T1 times at 12'. Model 1 included covariates glomerular filtration rate, pre-contrast T1 values adjusted to the heart rate, time difference between gadolinium injection and imaging, and gadolinium dosage in mmol/kg. Model 2 included adjustment for age, gender and ethnicity in addition to model 1 covariates.

Diastolic function parameter	Coefficient (p value)		
	Univariate	Model 1	Model 2

E peak	4.6E-4 (0.125)	3.4E-4 (0.311)	6.5E-4 (0.073)

SRI	-1.04E-3 (0.021)	-1.14E-3 (0.014)	-1.15E-3 (0.022)

## Conclusions

Diffuse myocardial fibrosis as assessed by decreased T1 times was associated with worse diastolic function as assessed by increased values of SRI. SRI as a diastolic function marker is more sensitive to the presence of myocardial fibrosis than the conventionally used Epeak.

## Funding

NHLBI grant - HL066075.

